# Metabolomics in Motion: Translating Molecular Signatures into Clinical Impact

**DOI:** 10.3390/metabo16030192

**Published:** 2026-03-12

**Authors:** Dimitris Kounatidis, Iordanis Mourouzis

**Affiliations:** 1 Diabetes Center, First Department of Propaedeutic Internal Medicine, Laiko General Hospital, Medical School, National and Kapodistrian University of Athens, 11527 Athens, Greece; 2 Department of Pharmacology, National and Kapodistrian University of Athens, 11527 Athens, Greece; imour@med.uoa.gr

Metabolomics, the comprehensive and quantitative analysis of small-molecule metabolites, is a rapidly advancing and expanding field within contemporary systems biology. By capturing both biochemical intermediates and the end-products of cellular processes, metabolomic profiling provides a direct and dynamic readout of metabolic activity [1]. Beyond their roles in primary metabolism and energy production, metabolic intermediates and end-products also function as critical regulators of cellular fate. Many serve as essential co-factors for epigenetic enzymes, while others modulate stem-cell proliferation and differentiation, shape the trajectory of cellular senescence, and contribute to the regulation of key kinase-driven signaling pathways [2,3].

Advances in mass spectrometry (MS), nuclear magnetic resonance (NMR) spectroscopy, and high-performance separation technologies have now enabled high-resolution, wide-coverage analysis of diverse metabolite classes, including lipids, amino acids (AAs), steroids, bile acids (BAs), and endocannabinoids (ECs) [4]. Collectively, these technological developments have positioned metabolomics as a powerful platform for biomarker discovery, mechanistic investigation, and therapeutic development. The Special Issue “Metabolomics in Human Diseases and Health” brings together nine contributions that highlight the expanding translational impact of this rapidly evolving field.

Among current areas of human health research, obesity remains a major focus, and metabolomics is emerging as a particularly promising tool. In a prospective study of 23 adults with obesity, Alfadda et al. used liquid chromatography coupled with high-resolution MS to investigate the metabolomic effects of treatment with the glucagon-like peptide 1 (GLP-1) receptor agonist liraglutide at the dose of 3 mg for twelve weeks. The study reported significant dysregulation of 161 endogenous metabolites. Most of these metabolites belonged to the class of oxidized lipids, phosphoglycerophosphates, N-acylated AAs, steroid hormones, and BAs. Pathway analyses revealed effects on inflammatory and metabolic signaling, including nuclear factor kappa-light-chain-enhancer of activated B cells (NF-κB), mitogen-activated protein kinase (MAPK), insulin pathways, and free radical scavenging processes. These findings support a role for liraglutide in reducing inflammation, improving lipid metabolism, and alleviating oxidative stress (Contribution 1). Beyond pharmacotherapy, substantial benefits in the management of obesity can also be achieved through metabolic bariatric surgery (MBS). In our narrative review, we showed that individuals with obesity who undergo MBS experience reductions in branched-chain amino acids (BCAAs), tryptophan, free fatty acids (FFAs), and ceramides, along with alterations in BAs, ECs, and microbiota-derived metabolites (Contribution 2).

The intestinal microbiome is a fast-developing area of investigation across all fields of human health and is recognized as a major determinant of metabolic and gastrointestinal function. In their review, Zheng et al. demonstrated that chronic constipation is influenced in part by shifts in microbial metabolites. Reductions in short-chain fatty acids (SCFAs) impair intestinal motility and promote inflammation, while disruptions in BAs, AAs, neurotransmitters, and microbial gas production further contribute to abnormalities in gastrointestinal transit (Contribution 3).

Metabolomics is also gaining traction in oncology, providing detailed insights that refine current models of tumor biology. Perestrelo and Luis highlighted how MS- and NMR-based profiling can reveal subtype-specific metabolic programs in breast cancer and identify candidate biomarkers relevant to diagnosis, prognosis, and treatment stratification. Their work further underscored key pathways such as glycolysis, lipid metabolism, and glutaminolysis, which appear central to tumor aggressiveness and resistance to therapy (Contribution 4). Potential benefits may also arise for prostate cancer. Kipura et al. developed a validated, semi-automated robotic extraction workflow and a rapid dual-column U(H)PLC-MRM-MS method for the absolute quantification of 20 AAs and six tryptophan metabolites with nanomolar limits of detection. This platform enabled the differentiation of treatment groups and the identification of metabolite patterns that distinguish healthy individuals from patients under active surveillance (Contribution 5).

Neuropsychiatric disorders, which are highly complex and heterogeneous and continue to rise in global prevalence, represent another important area for metabolomic investigation. A case–control study of 97 adults from Northern Italy linked chronic manganese exposure to Parkinsonism through distinctive metabolic and lipidomic signatures. Metabolites associated with the disease included 3-sulfoxy-L-tyrosine, formiminoglutamic acid, and glyoxylic acid, while exposure-related alterations were characterized by elevated glycocholic acid and disruptions in butanoate and glutamate metabolism. Pathway analyses additionally suggested perturbations in vitamin B6 metabolism, glucose regulation, ferroptosis, and EC signaling (Contribution 6). In schizophrenia, an exploratory work from the Hopkins First Episode Psychosis Project used both targeted and untargeted liquid chromatography–MS to analyze the cerebrospinal fluid (CSF) from individuals experiencing a first episode of psychosis (FEP). Although no metabolite remained significant after correction for multiple testing, the study identified several candidates that may reflect early biochemical abnormalities within the central nervous system. These included reduced levels of N-acetylneuraminic acid, N-acetyl-L-aspartic acid, L-phenylalanine, and 4-hydroxycinnamic acid, as well as increased levels of uric acid (Contribution 7).

Aging is one of the strongest determinants of disease susceptibility and is increasingly understood as a systemic metabolic process. In a comparative, metabolome-level analysis, Lokhov et al. demonstrated that aging shares the same metabolic pathways with pathological conditions, with an estimated probability of 99.96%. Interestingly, their work led to the construction of a metabolic metapathway that illustrates the biochemical convergence among aging, overall health status, and multimorbidity (Contribution 8). Despite the substantial benefits of metabolomics, major analytical challenges remain. Li et al. addressed issues related to missing data and small sample sizes by integrating mechanistic metabolic models with experimental, time-resolved metabolomics obtained from a meal-challenge test. Using coupled tensor factorizations, the researchers enhanced pattern discovery and strengthened correlations with body mass index (BMI)-related phenotypes, particularly in men, while also demonstrating the importance of accurate prior information for robust and reliable data integration (Contribution 9). Figure 1 presents the timeline of the articles included in this Special Issue.

The articles presented in this Special Issue highlight the considerable potential of metabolomics to deepen our understanding of diverse aspects of human health, including metabolic disorders such as obesity, cancer, and neuropsychiatric conditions. Despite these advances, the clinical translation of metabolomics remains constrained by several factors, including variability in sample collection and handling, heterogeneity in data acquisition methods, lack of standardized workflows, and a large fraction of unannotated spectral features, all of which limit reproducibility and biological interpretability. The high cost of instrumentation and the requirement for specialized expertise further restrict widespread accessibility (Contribution 2). Moving forward, progress will depend on the adoption of harmonized analytical protocols, expansion of large and well-designed phenotyped cohorts, and systematic clinical validation of findings. Innovations in high-resolution MS, NMR, and multi-omics integration, combined with artificial intelligence and machine learning for enhanced data analysis, are expected to expand metabolite coverage and improve predictive power. Stronger collaboration among clinicians, researchers, and regulatory agencies will be critical to translate metabolic signatures into actionable biomarkers and integrate metabolomics into precision medicine (Contribution 4).

## Figures and Tables

**Figure 1:**
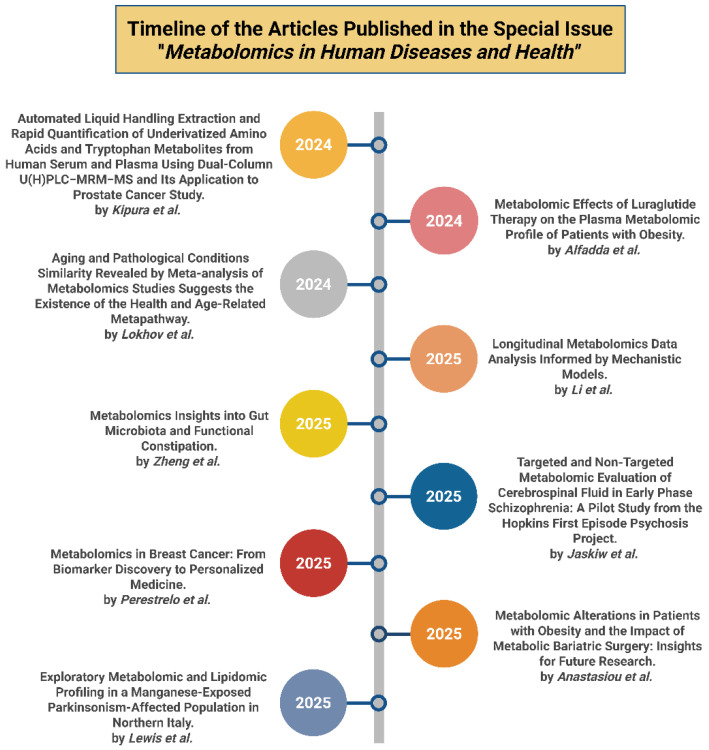
Timeline of the articles published in the Special Issue “Metabolomics in Human Diseases and Health” (Contributions 1–9). Created in BioRender. Kounatidis, D. (2026) https://BioRender.com/fvxw9pn (assessed on 20 February 2026).

## Data Availability

No new data were created or analyzed. Data sharing is not applicable.

## Abbreviations

The following abbreviations are used in this manuscript:

AA	amino acid
BA	bile acid
BCAA	branched-chain amino acid
BMI	body mass index
CSF	cerebrospinal fluid
EC	endocannabinoid
FEP	first episode of psychosis
FFA	free fatty acid
GLP-1	glucagon-like peptide 1
MAPK	mitogen-activated protein kinase
MBS	metabolic bariatric surgery
MS	mass spectrometry
NF-κB	nuclear factor kappa-light-chain-enhancer of activated B cells
NMR	nuclear magnetic resonance
SCFA	short-chain fatty acid
